# Comparison of Hemostatic Activity in Canine Leukoreduced Cryoprecipitate, Cryopoor Plasma, and Fresh Plasma

**DOI:** 10.1111/vcp.70017

**Published:** 2025-06-16

**Authors:** Roberta Perego, Eva Spada, Luciana Baggiani, Giuliano Ravasio, Enrica Zucca, Graziella Vanosi, Giancarlo Ruffo, Daniela Proverbio

**Affiliations:** ^1^ Veterinary Transfusion Research Laboratory (REVLab), Department of Veterinary Medicine and Animal Science (DIVAS) University of Milan Lodi Italy; ^2^ Department of Veterinary Medicine and Animal Science (DIVAS) University of Milan Lodi Italy

**Keywords:** blood components, dog, factor VIII, factor X, leukoreduction

## Abstract

**Background:**

To date, no studies have reported the evaluation of hemostatic activity in canine leukoreduced cryoprecipitate (LR‐CRYO) and leukoreduced cryopoor plasma (LR‐CPP).

**Objectives:**

We aimed to compare the hemostatic activity of LR‐CRYO and LR‐CPP to leukoreduced fresh plasma (LR‐FP) and to evaluate the preservation of LR‐CRYO by refrigeration and refreezing after thawing.

**Methods:**

Four hundred fifty milliliters of fresh blood was collected from ten donor dogs, leukoreduced, and separated into LR‐FP, then frozen (−20°C) to obtain leukoreduced fresh frozen plasma (LR‐FFP). LR‐FFP was further separated into LR‐CRYO and LR‐CPP. LR‐CRYO was frozen, thawed, and divided into two bags, one refrigerated for 24 h and one refrozen for 7 days. Factor VIII (FVIII) and X (FX) activity, prothrombin time, activated partial thromboplastin time, antithrombin III (ATIII) activity (ATA), total protein, albumin, fibrinogen, and D‐dimer concentration, and von Willebrand Factor (vWF) activity were measured in LR‐FP, LR‐CRYO, LR‐CPP, refrigerated, and refrozen LR‐CRYO.

**Results:**

FVIII activity was higher in LR‐CRYO (*p* = 0.0001) versus LR‐FP. vWF activity (*p* < 0.0001) and fibrinogen concentration (*p* = 0.0012) were lower in LR‐CPP versus LR‐FP. FX activity was higher in LR‐CPP (*p* < 0.0001) and LR‐FP (*p* = 0.0002) versus LR‐CRYO, and albumin concentration was higher in LR‐CPP versus LR‐FP (*p* < 0.0001) and LR‐CRYO (*p* < 0.0001). No statistically significant difference was found in refrigerated or refrozen LR‐CRYO as compared with LR‐CRYO, excluding ATA, which was lower (*p* = 0.0062) in refrigerated LR‐CRYO.

**Conclusions:**

Because the concentration of FVIII is higher in LR‐CRYO than in LR‐FP, LR‐CRYO is a possible component therapy when this factor is deficient. Since no statistically significant difference was found in refrozen LR‐CRYO as compared with LR‐CRYO, LR‐CRYO can be frozen after thawing for reuse.

## 1 Introduction

Current human and veterinary transfusion medicine favors goal‐oriented blood component selection based on individual patient requirements [1]. Cryoprecipitate (CRYO), a blood component that precipitates when fresh frozen plasma (FFP) is slowly, partially thawed and centrifuged [2], contains cold‐insoluble plasma proteins (i.e., von Willebrand factor [vWF], factor VIII [FVIII], factor XIII, fibrinogen, and fibronectin) [3]. Its key therapeutic advantage compared with FFP is higher hemostatic plasma protein concentration while in a much smaller plasma volume, thereby avoiding volume overload in recipients that are normovolemic or sustain congestive heart failure or renal failure [4]. CRYO might be used to treat coagulopathies in critically ill patients, but with the advent of recombinant human vWF and FVIII proteins, CRYO is now mainly used in humans in bleeding trauma patients requiring massive transfusion [1] and those with dysfibrinogenemia and acquired hypofibrinogenemia [5, 6, 7]. In dogs, CRYO is mainly used for bleeding management or prevention in cases of hemophilia A and von Willebrand disease (vWD) [4, 8]. In human medicine, CRYO preparations have been evaluated regarding their indications, dosing, and potential adverse effects [9, 10, 11, 12, 13]. Likely, canine CRYO has similar qualities to those in humans, although these have yet to be confirmed. The potential uses of CRYO in dogs were recently reviewed [1]. In vitro studies of canine CRYO are limited [2, 14, 15]. In one study, FVIII and vWF stabilities in canine CRYO were determined. Another, more recent study has examined the ability of cryopoor plasma (CPP) to correct vitamin K‐dependent coagulopathy in dogs.

The supernatant fraction, removed when producing CRYO, is termed cryopoor plasma (CPP), cryosupernatant, or cryodepleted plasma. It is considered a less useful by‐product component of CRYO production for treating hemostatic disorders. Nevertheless, recent studies in dogs suggest that CPP contains similar concentrations of vitamin K‐dependent factors II, VII, and X (FX) and higher albumin concentration and colloid osmotic pressure (COP) [14] than FFP. CPP improves the clotting times and vitamin K‐dependent coagulation factors concentrations similarly to FFP [16].

Whole blood or blood component transfusions might cause serious, potentially fatal adverse recipient reactions, some of which are caused by the presence and activity of leukocytes [17, 18]. Therefore, human blood components are subjected to leukoreduction [19]. In human blood components, leukoreduction might lead to decreased coagulation factors VII, VIII, and XI, and longer activated plasma thromboplastin time (aPTT) in leukoreduced FFP [20, 21, 22], although reported results vary [23, 24, 25], and differences between studies are possibly attributed to differences in filter type [21]. Nevertheless, none of the differences observed in leukoreduced components was clinically significant [22]. Recently, several in vitro veterinary studies have reported production with leukoreduction of canine blood components [17, 26, 27, 28, 29, 30, 31, 32, 33, 34, 35, 36, 37, 38] and their use [39, 40, 41, 42, 43, 44]. Only two studies [30, 38] have evaluated coagulation factor levels in canine leukoreduced FP, finding no or few significant ones (i.e., factor XI and aPTT) [30], which do not affect the component's therapeutic efficacy.

To the authors' knowledge, the hemostatic activity of canine leukoreduced CRYO and CPP has not been reported. The main aim of this study was therefore to determine the hemostatic activity of leukoreduced CRYO (LR‐CRYO) and leukoreduced CPP (LR‐CPP) compared to that of leukoreduced fresh plasma (LR‐FP). Unlike human blood components, there are no relevant differences in most hemostatic factors between leukoreduced and non‐leukoreduced canine plasma. We hypothesized that the hemostatic characteristics of canine LR‐CRYO and canine non‐leukoreduced CRYO are similar and that LR‐CRYO would show higher concentrations of fibrinogen, FVIII, and vWF levels than its source, LR‐FP, and LR‐CPP would show lower levels of these factors. We also hypothesized that LR‐CPP albumin concentration and FX activity would be similar to or higher than their levels in LR‐FP. A secondary aim of this study was to determine the hemostatic activity of LR‐CRYO stability when refrigerated and when refrozen after thawing.

## 2 Materials and Methods

### 2.1 Blood Donors

The study was approved by the Animal Welfare Bio Ethical Committee of the University of Milan (OPBA_26_2018). Blood units were collected with the dog owners informed consent from 10 blood donor dogs (intact females, 6; intact males, 4), with a mean age of 3.2 years (±1.03 years, range 2–5 years), of different breeds (Segugio Italiano, 4; Dogo Argentino, 4; Bernese mountain dogs, 2) belonging to the Volunteer Blood Donors Program of the Veterinary Transfusion Research Laboratory (REVLab) of the Department of Veterinary Medicine and Animal Science, University of Milan. The donors were DEA‐1 (Lab Test DEA 1, Alvedia, Limonest, France), DEA‐4 and Dal‐positive, and DEA‐7 negative (ID‐CARD NaCl enzyme test and cold agglutinins, DiaMed GmbH, Cressier FR, Switzerland). The donor dogs fulfilled the criteria for selection issued by the Italian Ministry of Health guidelines for veterinary transfusion medicine [45] (i.e., body weight > 25 kg, age 2–8 years, current vaccination, and ecto‐ and endo‐parasites prophylaxis). Dogs were deemed healthy based on their unremarkable clinical history, physical examination, complete blood count, routine serum biochemistry, and hemostatic tests (prothrombin time, activated partial thromboplastin time and fibrinogen concentration) and negative serology for *Dirofilaria immitis* (Snap Canine Heartworm antigen test kit, IDEXX), *Anaplasma phagocytophilum* (Fluo Anaplasma Biopronix kit, Agrolabo Spa, Scarmagno, Turin, Italy), *Ehrlichia canis* (Fluo Ehrlichia Biopronix kit Agrolabo Spa, Scarmagno, Turin, Italy), *Babesia canis* (Fluo Babesia Biopronix kit, Agrolabo Spa, Scarmagno, Turin, Italy), *Leishmania infantum* (Fluo Leishmania Biopronix kit, Agrolabo Spa, Scarmagno, Turin, Italy), *Rickettsia conorii* (Fluo Rickettia Biopronix kit, Agrolabo Spa, Scarmagno, Turin, Italy). In addition, for *Babesia* spp., microscopic observation of a fresh blood smear was performed, while for *Dirofilaria* spp. the microhematocrit method was done. Donors were not administered medications known to interfere with hemostasis for 6 months prior to the study.

### 2.2 Blood Collection and Processing

Whole blood (450 ± 50 mL) was collected into a commercial closed collection system for human use (CompoSelect Premium T&T CPD/SAG‐M, Fresenius Kabi Italia S.r.l., Isola della Scala (VR) Italy) by jugular or cephalic venipuncture by gravity with the help of a blood collection scale/mixer with high/low blood flow alarm (Hemotek‐2, Delcon, Italy) with no sedation, following standard operating procedures of the Veterinary Transfusion Research Laboratory (REVLab) of the Department of Veterinary Medicine and Animal Science, University of Milan. No adverse reactions were noted in dogs during or after blood donation. The blood collection quadruple bag system, previously shown to be applicable for use in dogs [27], consisted of a primary bag, containing 63 mL of citrate, phosphate, dextrose (CPD) anticoagulant‐preservative solution in the and 100 mL of sodium chloride‐adenine‐glucose‐mannitol (SAG‐M) solution in one of the two satellite storage bags. The primary bag was connected to a secondary empty bag with an in‐line leukocyte soft filter for pre‐storage leukoreduction. This filter is a neutral‐charged, third‐generation polyester filter, fabricated to remove both leukocytes (WBCs) and platelets (PLTs). After collection, the primary bag containing the collected blood was macroscopically inspected for clots, delicately mixed, weighed, and a 2 mL aliquot was taken for further analysis. The integral canal above the filter was then broken, and the contents of the primary bag were drained through the filter and collected by gravity into the secondary bag at room temperature (20°C). Filtration time varied between 15 and 30 min. All 10 leukoreduced whole blood (LR‐WB) units were collected on the same day and stored at 2°C–6°C pending completion of collections.

After leukoreduction, a 2 mL aliquot of LR‐WB was taken from each unit for subsequent analysis. The secondary bags, containing LR‐WB, were then centrifuged (4657 *g* for 15 min, 4°C; Rotixa 50RS centrifuge, Hettich Lab Technology, Milan, Italy) within 6 h from blood collection. The supernatant leukoreduced fresh plasma (LR‐FP) was expressed into an attached empty satellite bag using a manual plasma extractor, leaving approximately 2 mL of LR‐FP in the transfer tubing of the satellite bag, which was transferred into a polypropylene test tube for further analysis. The LR‐FP bag (containing ~200 mL) was separated from the collection system and from the transfer tubing using an electric tube sealer (HemoWeld, Delcon, Monza Brianza, Italy) and frozen at −20°C in a dedicated plasma unit freezer (Fiocchetti snc, Luzzara, Italy) to prepare LR‐FFP, used in this study. The remaining leukoreduced packed RBCs were transferred into the SAG‐M satellite bag and stored at 2°C–6°C in a dedicated blood unit refrigerator (Fiocchetti snc, Luzzara, Italy) and were used for transfusion purposes unrelated to this study. Within 2 days of freezing at −20°C, the LR‐FFP was thawed in a refrigerator at 2°C–6°C for 15–18 h, until reaching a slushy consistency, and then centrifuged (at 1°C–6°C, at 4657 *g* for 5 min), as previously described [14]. The precipitate product obtained was the leukoreduced cryoprecipitate (LR‐CRYO) while the supernatant fraction was the leukoreduced cryopoor plasma (LR‐CPP), which was then transferred into a satellite bag (Compoflex Transfer Bag, 150 mL) with a sterile spike (Fresenius Kabi Italia S.r.l., Isola della Scala, VR, Italy), while the LR‐CRYO (~ 60–90 mL) remained in the principal bag. LR‐CCP and LR‐CRYO 2 mL aliquots were collected for further analysis.

For the second purpose of the study, before freezing, LR‐CRYO was divided into two satellite plasma bags containing 30–40 mL volumes using a sterile spike (Fresenius Kabi Italia S.r.l., Isola della Scala, VR, Italy). After 24 h, the two frozen LR‐CRYO satellite bags were thawed in a water bath at 37°C, and one bag was refrigerated at 2°C–6°C (refrigerated‐LR‐CRYO) while the other bag was refrozen at −20°C (refrozen‐LR‐CRYO). After 24 h, a 2 mL refrigerated‐LR‐CRYO aliquot was obtained for further analysis. After 7 days, the refrozen‐LR‐CRYO bag was rethawed in a water bath at 37°C, and then, similarly, a 2 mL aliquot was obtained for further analysis. Finally, a 2 mL aliquot was obtained from the transfer tubing of the refrozen‐LR‐CRYO bag (tube‐refrozen‐LR‐CRYO) to compare with the results of the analysis done in the refrozen‐LR‐CRYO bag sample (refrozen‐LR‐CRYO) to determine, for future studies, if the transfer tubing content adequately represents the bag content.

### 2.3 Laboratory Analysis

**FIGURE 1:**
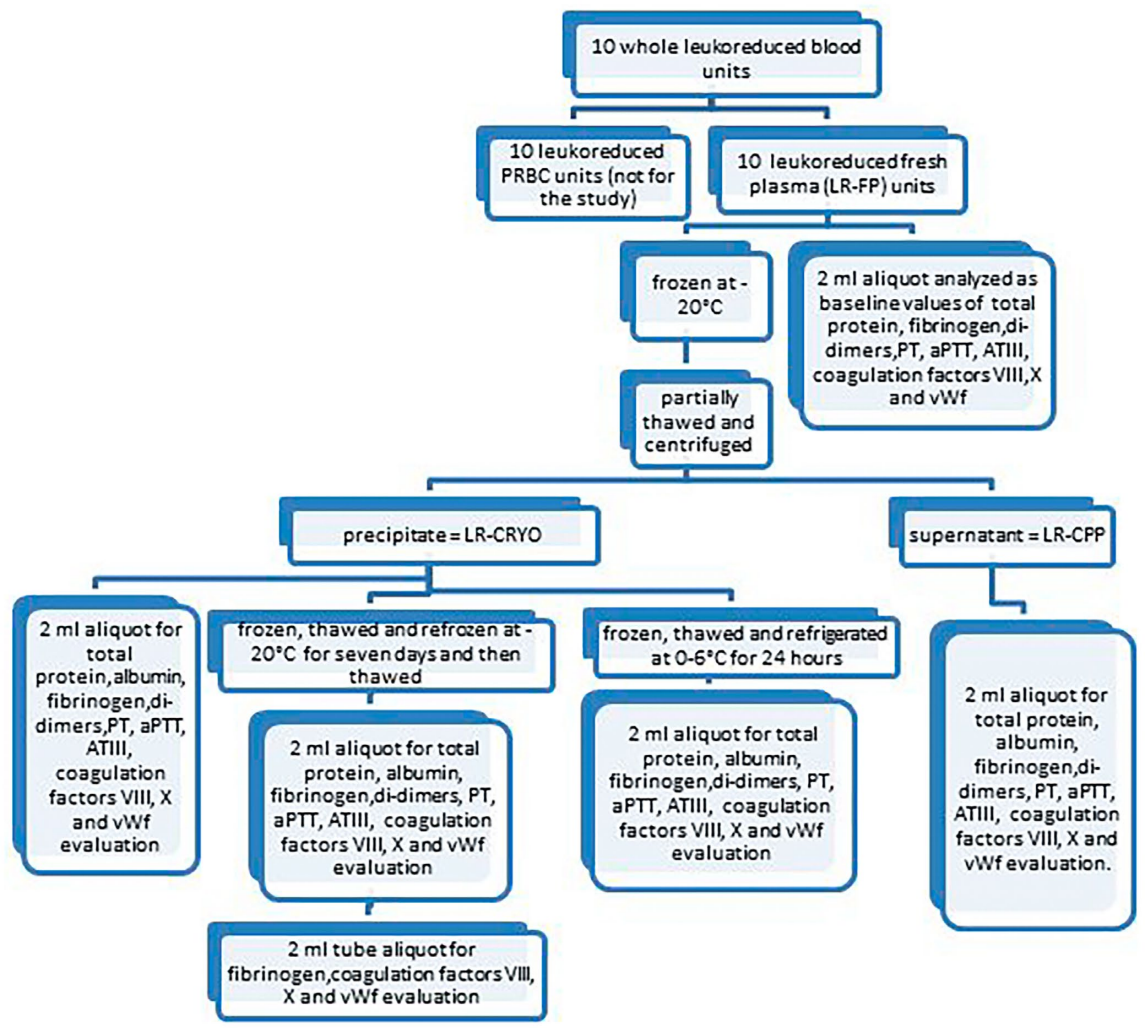
Schematic flowchart of study design.

The sample aliquots of WB, LR‐WB, LR‐FP, LR‐CRYO, LR‐CPP, refrigerated‐LR‐CRYO, refrozen‐LR‐CRYO, and tube‐refrozen‐LR‐CRYO were all placed into polypropylene test tubes containing no anticoagulant and analyzed immediately after sampling. For technical reasons, WB and LR‐WB CBC (Cell‐Dyn 3500—Abbott S.r.l., Italy) were performed in only 6/10 samples. With the exception of tube‐refrozen‐LR‐CRYO, the parameters assessed from each sample included factor VIII (FVIII), factor X (FX), antithrombin III (ATIII), and von Willebrand factor (vWF) activities, prothrombin time (PT), activated partial thromboplastin time (aPTT), and fibrinogen, D‐Dimers (DD), total protein, and albumin concentrations. In the tube‐refrozen‐LR‐CRYO samples, only FVIII, FX, and vWF activities and fibrinogen concentration were measured (Figure 1).

Hemostatic tests, except fibrinogen evaluation, were measured using a coagulometric analyzer (STA Compact Max, Stago Italia SRL, Milan, Italy) with commercial reagents obtained from the analyzer manufacturer. Fibrinogen concentration was measured using the Clauss method (Hagen Diagnostica S.r.l., San Giovanni Valdarno, Italy), total protein (TP, Biuret method, Hagen Diagnostica S.r.l., Firenze, Italy), and albumin (bromocresol green method, Hagen Diagnostica S.r.l., Firenze, Italy) concentrations were measured using spectrochemistry (Cobas Mira Classics, Roche Diagnostics, Basilea, Switzerland). FVIII and FX activities were measured with pooled canine citrate plasma obtained from 10 healthy dogs as a control. For DD, PT, and aPTT, the calibration curve was imported by bar code at each new batch. For ATIII and vWF activities, commercial human plasma controls were used (STA‐Unicalibrator e STA‐vWF: Ag calibrator, Diagnostica Stago, Asnières‐sur‐Seine, France).

### 2.4 Statistical Analysis

The distribution pattern of results was determined using the Kolmogorov–Smirnov test. Analyte in different sample types (i.e., LR‐FP, LR‐CRYO, LR‐CPP, and refrigerated‐LR‐CRYO and refrozen‐LR‐CRYO) was compared using ANOVA, with Bonferroni's correction for multiple comparisons, followed by post hoc paired Student's *t*‐test or Wilcoxon Rank‐Signed test, depending on the data distribution pattern. All the tests were two‐tailed, and in all, *p* < 0.05 was considered significant. *T*‐test or Wilcoxon test, depending on the data distribution, was used to compare WB and LR‐WB groups and refrozen‐LR‐CRYO and tube‐refrozen‐LR‐CRYO groups. Statistical analyses were conducted, and graphs were created using a commercial software package (MedCalc Statistical Software version 20.027, MedCalc Software Ltd., Ostend, Belgium).

## 3 Results

The only significant differences in CBC analytes between WB and LR‐WB were in the leukocyte count (10.6 ± 3.6 × 103/μL vs. 0.2 ± 0.1 × 103/μL, respectively; *p* = 0.0009) and platelet count (22 ± 54 × 103/μL vs. 44 ± 22 × 103/μL, respectively; *p* = 0.0002). The PT, aPTT, FVIII, FX, AT, and vWF activities, and fibrinogen, DD, albumin, and TP in LR‐FP, LR‐CRYO, and LR‐CPP are reported in Table 2. FVIII activity was higher (*p* = 0.0001) in LR‐CRYO and lower (*p* = 0.0003) in LR‐CPP compared with LR‐FP. FX activity was lower in LR‐CRYO compared with LR‐FP (*p* = 0.0002) and LR‐CPP (*p* < 0.0001). vWF activity was lower in LR‐CPP compared with LR‐CRYO (*p* = 0.0217) and LR‐FP (*p* < 0.0001) and was insignificantly different between LR‐CRYO and LR‐FP (*p* = 0.3336).

**FIGURE 2:**
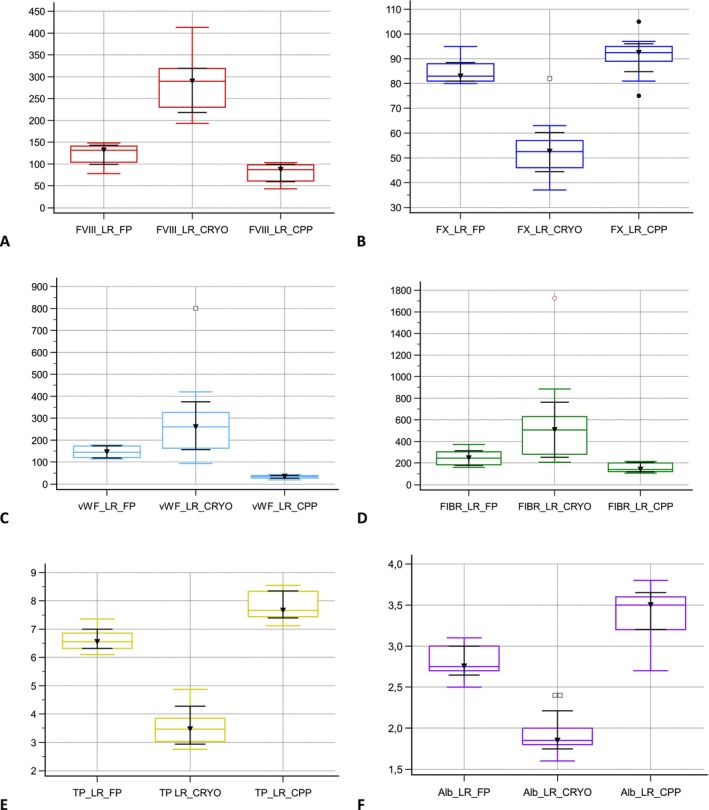
Box and whisker plots of some activity levels and concentrations measured for each unit of LR‐fresh plasma (LR‐FP), LR‐cryoprecipitate (LR‐CRYO) and LR‐cryopoor plasma (LR‐CPP). (A) Factor VIII activity levels, Y‐axis represent %, (B) factor X activity levels, Y‐axis represent %, (C) Von Willbrand VIII activity levels, Y‐axis represent %, (D) fibrinogen concentrations, Y‐axis represent mg/dL, (E) total protein concentrations, Y‐axis represent g/L, and (F) albumin concentrations, Y‐axis represent g/L.

Median fibrinogen concentrations for LR‐FP, LR‐CPP, and LR‐CRYO were 251.6 mg/mL (min–max, 160.3–370.6), 152.4 mg/mL (min–max, 106–214.7), and 506 mg/mL (min–max, 207.2–1727), respectively (Table 1, Figure 2). Fibrinogen concentrations were higher in LR‐FP compared with LR‐CPP (*p* = 0.0012) but were not different between LR‐CRYO and LR‐CPP (*p* = 0.3847) or LR‐FP (*p* = 0.1451) (Figure 2).

**TABLE 1:** Descriptive statistics relative to hemostatic activity in each hemocomponent.

Analyte (RI)	Component	Min	Max	Median (95% CI)	*p‐*value versus LR‐FP	*p* versus LR‐CRYO
PT (7–9.8 s)	LR‐FP	7.3	8.7	8.1 (7.6–8.4)	—	**0.0202**
	LR‐CRYO	7.6	9.5	8.6 (8.3–9.3)	**0.0202**	—
	LR‐CPP	8.6	10.4^a^	9.6 (8.9–10.1)	**0.0001**	**0.0182**
aPTT (11–16.9 s)	LR‐FP	13.4	15.6	14.6 (13.9–15)	—	**0.0094**
	LR‐CRYO	15.4	24.6^a^	18.5 (17.3–21.2)^a^	**0.0094**	—
	LR‐CPP	12.7	20.1^a^	14.6 (14–15.7)	1.000	**0.0021**
ATIII (95%–140%)	LR‐FP	112	132	128 (119–132)	—	**0.0008**
	LR‐CRYO	68^a^	127	88 (76–105)^a^	**0.0008**	—
	LR‐CPP	121	136	*136 (133–136)*	**0.0208**	**0.0006**
FVIII (50%–150%)	LR‐FP	78	148	132 (104–141)	—	**0.0001**
	LR‐CRYO	193^a^	413^a^	289 (230–319)^a^	**0.0001**	—
	LR‐CPP	43^a^	103	87 (61–98)	**0.0003**	**0.0001**
FX (80%–175%)	LR‐FP	80	95	83 (81–88)	—	**0.0002**
	LR‐CRYO	37	82	53 (46–57)^a^	**0.0002**	—
	LR‐CPP	75	105	93 (89–95)	0.4055	**< 0.0001**
vWF (70%–180%)	LR‐FP	114	178	145 (121–173)	—	0.3336
	LR‐CRYO	94	801^a^	261 (163–326)^a^	0.3336	—
	LR‐CPP	19^a^	43^a^	34 (27–39)^a^	**< 0.0001**	**0.0217**
Fibrinogen (100–400 mg/mL)	LR‐FP	160	371	245 (184–304)	—	0.3847
	LR‐CRYO	207	1727^a^	*506 (254–763.9)* ^a^	0.3847	—
	LR‐CPP	106	215	140.6 (120.7–201)	**0.0012**	0.1451
Total protein (6–8 g/dL)	LR‐FP	6.1	7.4	6.6 (6.3–6.9)	—	**< 0.0001**
	LR‐CRYO	2.8^a^	4.9^a^	3.5 (3–3.9)^a^	**< 0.0001**	—
	LR‐CPP	7.1	8.6^a^	7.7 (7.4–8.3)	**< 0.0001**	**< 0.0001**
Albumin (2.3–3.2 g/dL)	LR‐FP	2.5	3.1	2.8 (2.7–3)	—	**0.0001**
	LR‐CRYO	1.6^a^	2.4	1.9 (1.8–2)^a^	**0.0001**	—
	LR‐CPP	2.7	3.8^a^	3.5 (3.2–3.6)^a^	**< 0.0001**	**< 0.0001**
D‐Dimers (0.01–0.35 μg/mL)	LR‐FP	0.12	0.27	0.16 (0.15–0.18)	—	1.0000
	LR‐CRYO	0.05	0.75^a^	*0.16 (0.09–0.49)*	1.0000	—
	LR‐CPP	0.15	1.25^a^	*0.26 (0.17–0.57)*	0.7507	0.1926

*Note:* Analytes with *non‐normal distribution* are reported in *italics*. Bold values are statistically significant.

Abbreviations: aPTT, activated partial thromboplastin time; ATIII, antithrombin III; CI, confidence interval; FVIII, factor VIII; FX, factor X; LR‐CPP, leukoreduced cryopoor plasma; LR‐CRYO, leukoreduced cryoprecipitate; LR‐FP, leukoreduced fresh plasma; Max, maximum; Min, minimum; PT, prothrombin time; RI, reference interval; SD, standard deviation; vWF, factor von Willebrand.

^a^ Values are outside the reference range.

TP concentration was higher in LR‐CPP (median, 7.7 g/dL; min–max, 7.4–8.3) than in LR‐FP (median, 6.6 g/dL; min–max, 6.3–6.9) (*p* < 0.0001) and then in LR‐CRYO (median, 3.6 g/dL; min–max, 3–3.9) (*p* < 0.0001) (Table 1, Figure 2). LR‐FP TP was higher (*p* < 0.0001) compared with LR‐CRYO. Albumin concentration was higher in LR‐CPP (median, 3.5 g/dL; min–max, 3.2–3.6) than in LR‐FP (median, 2.8 g/dL; min–max, 2.7–3) (*p* < 0.0001) and then in LR‐CRYO (median, 1.9 g/dL; min–max, 1.8–2) (*p* < 0.0001). LR‐FP albumin was higher (*p* = 0.0001) compared with LR‐CRYO (Table 1, Figure 2).

**FIGURE 3:**
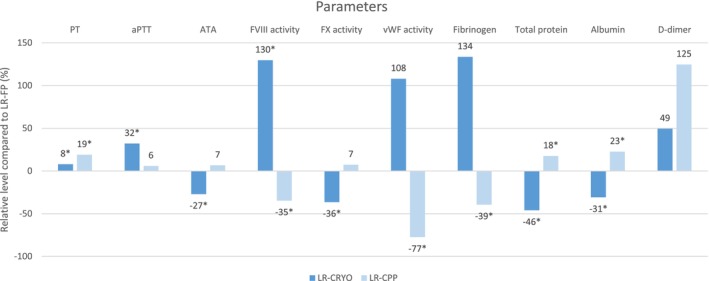
Mean relative difference in analyte levels in leukoreduced cryoprecipitate (LR‐CRYO) and leukoreduced cryopoor plasma (LR‐CPP) from leukoreduced fresh plasma (LR‐FP) depicted as baseline values. PT: prothrombin time; aPTT: activated partial thromboplastin time; FVIII: factor VIII; FX: factor X; ATA: antithrombin III activity; vWF: factor von Willebrand. Asterisks* denote significant difference (*p* < 0.05) compared with LR‐FP level.

The relative analyte level differences in LR‐CRYO and LR‐CPP from the LR‐FP baseline are illustrated graphically in Figure 3.

The only significant analyte difference between refrigerated‐LR‐CRYO and refrozen‐LR‐CRYO was in the ATA, which was lower (*p* = 0.0062) in refrigerated‐LR‐CRYO (median, 84%; min–max, 73–100) than in LR‐CRYO (median, 88%; min–max, 76–105) (Table 2).

**TABLE 2:** ANOVA test for repeated measures with Bonferroni correction to evaluate differences between LR‐CRYO, refrigerated‐LR‐CRYO, and refrozen‐LR‐CRYO groups.

Analyte (reference range)	Component	Min	Max	Mean (±SD) or median (95% CI)	*p* versus LR‐CRYO
PT (7–9.8 s)	LR‐CRYO	7.6	9.5	8.6 (8.3–9.3)	**—**
	Refrigerated‐LR‐CRYO	7.7	9.1	8.4 (8.1–8.5)	0.1133
	Refrozen‐LR‐CRYO	7.8	9.0	8.4 (8.1–8.8)	1.0000
aPPT (11–16.9 s)	LR‐CRYO	15.4	24.6^a^	18.5 (17.3–21.2)^a^	**—**
	Refrigerated‐LR‐CRYO	15.3	24.9^a^	18.3 (17–22)^a^	1.0000
	Refrozen‐LR‐CRYO	15.2	24.2^a^	19 (18–20.9)^a^	1.0000
ATIII (95%–140%)	LR‐CRYO	68^a^	127	88 (76–105)^a^	**—**
	Refrigerated‐LR‐CRYO	69^a^	123	84 (73–100)^a^	**0.0062**
	Refrozen‐LR‐CRYO	73^a^	115	89 (76–98)^a^	1.0000
FVIII (50%–150%)	LR‐CRYO	193^a^	413^a^	289 (230–319)^a^	**—**
	Refrigerated‐LR‐CRYO	114^a^	504^a^	232 (217–290)^a^	1.0000
	Refrozen‐LR‐CRYO	171^a^	407^a^	*222.5 (200.9–256.1)* ^a^	0.9009
FX (80%–175%)	LR‐CRYO	37	82	53 (46–57)^a^	**—**
	Refrigerated‐LR‐CRYO	41^a^	77	53 (52–54)^a^	1.0000
	Refrozen‐LR‐CRYO	41^a^	79^a^	55 (51–57)^a^	1.0000
vWF (70%–180%)	LR‐CRYO	94	801^a^	261 (163–326)^a^	**—**
	Refrigerated‐LR‐CRYO	100	810^a^	238 (138–340)^a^	0.9634
	Refrozen‐LR‐CRYO	51^a^	794^a^	*177 (141.4–310.9)*	0.1185
Fibrinogen (100–400 mg/mL)	LR‐CRYO	207.2	1727.4^a^	*506.2 (254–763.9)* ^a^	**—**
	Refrigerated‐LR‐CRYO	197.1	1984.2^a^	*457 (227.2–699.5)* ^a^	1.0000
	Refrozen‐LR‐CRYO	161.8	1998.0^a^	*345.45 (225.3–668.6)*	1.0000
Total protein (6–8 g/dL)	LR‐CRYO	2.8^a^	4.9^a^	3.5 (3–3.9)^a^	**—**
	Refrigerated‐LR‐CRYO	2.9^a^	4.7	3.5 (3.2–3.8)^a^	1.0000
	Refrozen‐LR‐CRYO	2.9^a^	4.7	3.6 (3.1–3.8)^a^	1.0000
Albumin (2.3–3.2 g/dL)	LR‐CRYO	1.6^a^	2.4	1.9 (1.8–2)^a^	**—**
	Refrigerated‐LR‐CRYO	1.6^a^	2.4	1.9 (1.7–2)^a^	1.0000
	Refrozen‐LR‐CRYO	1.6^a^	2.4	1.9 (1.7–2.1)^a^	1.0000
D‐Dimers (0.01–0.35 μg/mL)	LR‐CRYO	0.05	0.75^a^	*0.16 (0.09–0.49)*	**—**
	Refrigerated‐LR‐CRYO	0.07	0.77^a^	*0.21 (0.17–0.48)*	0.7469
	Refrozen‐LR‐CRYO	0.04	0.85^a^	*0.16 (0.1–0.5)*	1.0000

*Note:* Analytes with *nonnormal distribution* are reported in *italics*. Bold values are statistically signified.

Abbreviations: aPTT, activated partial thromboplastin time; ATIII, antithrombin III; CI, confidence interval; FVIII, factor VIII; FX, factor X; LR‐CRYO, leukoreduced cryoprecipitate; max, maximum; Min, minimum; PT, prothrombin time; refrigerated‐LR‐CRYO, refrigerated leukoreduced cryoprecipitate; refrozen LR‐CRYO, refrozen leukoreduced cryoprecipitate; SD, standard deviation; vWF, factor von Willebrand.

^a^ Values are outside the reference range.

The only significant analyte difference between refrozen LR‐CRYO and tube‐refrozen LR‐CRYO was in FX activity, which was higher (*p* = 0.0018) in tube‐refrozen LR‐CRYO (median, 59%; min–max, 57–63) than in refrozen LR‐CRYO (median, 55%; min–max, 51–57).

## 4 Discussion

To the author's knowledge, this study is the first reporting the PT, aPTT, ATA, FVIII, and FX activities, and DD, TP, and albumin concentrations in canine LR‐CRYO and LR‐CPP, comparing them to those of the source LR‐FP, and obtaining that FVIII activity was higher in LR‐CRYO vs. LR‐FP, FX activity was higher in LR‐CPP and LR‐FP vs. LR‐CRYO, and albumin concentration was higher in LR‐CPP vs. LR‐FP and LR‐CRYO.

Processing WB to obtain LR‐CRYO for transfusion requires a series of steps that might influence the stability of clotting factors. First, the filters used for leukoreduction might impact WB‐derived plasma quality. Studies in human medicine examined the effects of WB or plasma leukofiltration on residual coagulation factor activity, with conflicting results. Several studies that evaluated several leukoreduction filters in human fresh plasma units indicated that leukofiltration did not activate coagulation and affected coagulation factors activities [23, 24, 46, 47, 48, 49, 50]. Nevertheless, in other studies, during filtration, contact between artificial surfaces and plasma coagulation proteins might induce changes in clotting times and coagulation factor concentration and activity (significant losses of factors V, VII, VIII, IX, XI, and XII, decrease of fibrinogen levels, and increases in coagulation activation markers) [20, 21, 51].

In recent years, leukoreduction has become increasingly routine in veterinary transfusion medicine, with several studies of the use of various human [17, 26, 31, 32, 33, 34, 35, 36, 37, 38, 52] or veterinary [28, 29, 30] blood collection systems and leukoreduction filters. Nonetheless, few studies focused on leukoreduced plasma and coagulation factor activity [30, 38]. One study examined the PT, aPTT, fibrinogen, and DD concentrations, activity of coagulation factors V, VIII, X, XI, as well as ATA and vWF activity in canine leukoreduced FFP, produced using a veterinary collection and filtration system. The only statistically significant difference between LR‐FPP and non‐leukoreduced FFP included a longer aPTT and lower factor XI activity in the former [30]. Another recent study of canine LR‐FP and non‐LR‐FP produced from non‐precooled WB, employing a human blood collection device and a polyurethane leukocyte filter, similar to the one used in this study showed no significant differences between components in PT, aPTT, activity of coagulation factors V, VII, VIII, X, and XI, fibrinogen concentration and activity of AT and protein C [38]. Differences between results of previous studies possibly occurred due to differences in filter type [20, 24, 53] and WB storage temperature [26, 49, 53] or time‐period [21] prior to leukoreduction. In our study, we used a human leukoreduction system previously used in dogs [27, 34] which proved highly efficient, evident by markedly decreased leukocytes and platelets counts in LR‐WB compared with WB.

In the LR‐FP freezing and partial thawing production process, separation of plasma protein based on their freezing temperature, in theory, separates fibrinogen, FVIII, and vWF into the LR‐CRYO fraction, while other coagulation factors remain, at higher concentrations, in the LR‐CPP component [54]. Our results indicate that the method and the materials used in this study produced the predicted product with partial success, higher FVIII activity in a smaller plasma volume in LR‐CRYO compared with LR‐FP (approximately 60–90 mL compared with 200 mL, respectively) and higher (nearly double), albeit insignificantly, vWF activity and fibrinogen concentration compared with LR‐FP. The level of all these factors was higher in LR‐CRYO and LR‐FP as compared with LR‐CPP. The present results are somewhat similar to previous ones [14], with the advantage of the leukoreduction, although in the previous study vWF activity and fibrinogen concentration were, in fact, significantly higher in CRYO than in FFP and CCP. The absence of a statistically significant increase in LR‐CRYO vWF activity and fibrinogen concentration vs. LR‐FP in our study is possibly due to the small cohort with low statistical power and high individual variability of these factors LR‐CRYO levels, regardless of similar LR‐FP levels. Such high individual variability of fibrinogen concentration and FVIII and vWF activities in canine FPP and in its CRYO product is known in human donors, even between same‐donor samples obtained at different times [55, 56]. Furthermore, a recent study of the impact of hemostatic protein levels on CRYO quality has suggested the latter depends both on specific donor factors and the blood component production techniques used [2].

The use of CPP, though non‐leukoreduced, in dogs was reported in a single previous study focused on its impact on albumin concentration and colloid osmotic pressure in critically ill dogs with hypoalbuminemia [40]. Human CPP has lower levels of vWF, fibrinogen, FVIII, factor XIII, and fibronectin, but similar concentrations of other plasma proteins and clotting factors as the original FPP [3, 57]. CPP is used in place of FFP in human medicine primarily for the treatment of thrombotic thrombocytopenic purpura [58, 59], a condition not reported in dogs. CPP has also been used in human patients instead of FFP for the treatment of generalized peritonitis and disseminated intravascular coagulation associated with pancreonecrosis [60, 61]. In dogs, CPP was used for treating vitamin K antagonist rodenticide toxicity [40], and its effectiveness in that study is supported by our findings that LR‐CPP FX activity is to that of LR‐FP and higher than in LR‐CRYO, warranting further studies.

Hypoalbuminemia is common in various conditions in dogs (e.g., hemorrhage, septic peritonitis, protein‐losing nephropathy, and enteropathy) and is associated with death and poor healing [62, 63, 64]. Species‐specific albumin is the ideal replacement product for treating hypoalbuminemia, but canine‐specific albumin concentrate is unavailable, and therefore, in severe hypoalbuminemia, human serum albumin or synthetic colloids are administered, with their associated risks [65]. An alternative could be FFP, but the large volume of FFP needed to treat hypoalbuminemia can result in volume overload [66]. A recent publication [40] suggests the use of CPP for albumin replacement: our study demonstrated that total protein and albumin concentrations, as measured, were significantly higher in LR‐CPP compared with LR‐FP and LR‐CRYO, and confirms the results obtained on non‐leukoreduced CPP [40]. Therefore, CPP is an effective source of albumin, and thus, although hypoalbuminemia might be treated with canine plasma (fresh or not), CPP should be considered in such cases, in clinical settings where CRYO is routinely produced from FFP.

In this study, ATIII was found in lower concentrations in LR‐CRYO compared with LR‐FP, and this data is in line with what has been reported in some human studies on CRYO [67, 68] but contrasts with an older human study that reported no difference in ATIII content between FFP, CRYO, and CPP [69]. Conversely, it seems that CPP is an adequate ATIII source to be considered in cases of AT consumption and loss (e.g., acute pancreatitis, PLE and PLN).

In regard to clotting times, PT and aPTT values were increased in LR‐CRYO compared with LR‐FP, and these results might be related to the different concentrations of factor X in these products. In fact, factor X deficiency in humans is usually suspected when both plasma PT and aPTT are abnormal [70], and in LR‐CRYO the factor X concentration was significantly lower than in LR‐FP. Nonetheless, in our study, PT is more prolonged in CPP than in FP and CRYO, although FX activity is actually higher in CPP vs. the other components; therefore, the real reasons for this difference in clotting times remain unclear.

In human CRYO, fibrinogen and vWF are stable when refrigerated for 24 h after thawing [71]. A recent study demonstrated that thawed CRYO storage time extension up to 120 h, at either 2°C–6°C or at room temperature, is feasible and maintains FVIII, fibrinogen, and vWF international standard levels, at no risk of potential bacterial contamination [72]. Fibrinogen has been proven stable in CRYO, refrigerated, or stored at room temperature for 35 days after thawing, which nevertheless showed that FVIII and VWF levels decline significantly under both these conditions [73]. Canine and feline coagulation factors, fibrinogen, and vWF were shown to remain stable in FFP subjected to freezing and thawing cycles [74], while an old study in greyhound dogs indicates that CRYO, subjected to freezing and thawing cycles, maintains FVII activities and vWF concentrations similar to those in the original thawed precipitate [15]. The present study similarly confirms that in LR‐CRYO, refrigerated for 24 h or when refrozen for 7 days, all hemostatic analytes measured remained unchanged, excluding ATA, which was significantly lower in refrigerated LR‐CRYO as compared with LR‐CRYO. This decrease in ATA was an unexpected result that cannot be explained in the authors' opinion. Our results regarding the stability of LR‐CRYO suggest that thawed LR‐CRYO, when refrigerated for 24 h or refrozen for up to 7 days, can be efficacious if used for its intended indications, increasing the efficiency of blood component banking.

Finally, in this study, there was no significant difference in hemostatic analytes between refrozen LR‐CRYO bag and refrozen LR‐CRYO transfer tubing, excluding FX activity that was significantly higher, but not clinically significant, in the tubing compared with the bag. This was perhaps due to a more rapid freezing and thawing process in the transfer tubing, warranting further studies to prove this hypothesis. These data are important for future studies regarding refrozen LR‐CRYO long‐term storage, where in such studies a segment of the transfer tubing can be used to measure hemostatic analytes that adequately reflect their levels in the bag (excluding FX activity), avoiding the need to thaw the bag contents.

Despite the attentiveness to the study protocol, this study has some limitations. The major limitation is the limited sample size, which increases the type‐II error probability. The possibility that certain undetected filtration and processing effects did impact the hemostatic analyte results precludes the identification of minor group differences due to the limited sample size. Nonetheless, the present sample size is similar to those in previous veterinary studies of plasma, ranging between 8 and 15 [14, 15, 16].

Another limitation of this study is the failure to determine the hemostatic analytes in LR‐FFP, in addition to LR‐FP. This shortcoming is because no transfer tubing segment was available to measure LR‐FFP analytes prior to its LR‐CRYO production processing. Canine LR‐FPP hemostatic characteristics were reported [30, 38]; however, in the present study, a different filtration was used, and different leukoreduction filter types have different effects on hemostatic analytes [20]. Use of the same filtration system would have allowed a more complete comparison of the current findings of LR‐FFP hemostatic analytes with previous results. Therefore, the present results should be best applied to the specific filter used in this study and should be applied cautiously to other filtering systems. In addition, although some standard veterinary plasma processing protocols for processing exist [75, 76], differences in such protocols might preclude extrapolation of the results of this study to blood products produced following different protocols.

Lastly, this study did not examine the effect of long‐term LR‐CPP storage, warranting future studies. Nevertheless, under standard storage conditions, a LR‐CPP shelf‐life of up to 6 years is recommended by certain blood banks [14].

## 5 Conclusions

The results of this study support the use of canine LR‐CRYO for FVIII, fibrinogen, and vWF deficiencies, as previously suggested for canine non‐leukoreduced CRYO. The results also suggest that unused LR‐CRYO can be refrozen, stored, and re‐thawed for efficient use. LR‐CPP has high albumin concentration and FX activity, making it suitable for hypoalbuminemic dogs and those in need of FX.

## Conflicts of Interest

The authors declare no conflicts of interest.

## References

[1] J. Prittie , “The Role of Cryoprecipitate in Human and Canine Transfusion Medicine,” Journal of Veterinary Emergency and Critical Care 31, no. 2 (2021): 1–11, 10.1111/vec.13034. 33751762

[2] M. Drinkhouse , M. B. Brooks , D. Stefanovski , K. Marryott , and M. B. Callan , “Influence of Canine Donor Plasma Hemostatic Protein Concentration on Quality of Cryoprecipitate,” Journal of Veterinary Internal Medicine 33, no. 1 (2019): 124–131, 10.1111/jvim.15376. 30548342 PMC6335516

[3] R. L. Sparrow , D. W. Greening , and R. J. Simpson , “A Protocol for the Preparation of Cryoprecipitate and Cryodepleted Plasma,” Methods in Molecular Biology 728 (2011): 259–265, 10.1007/978-1-61779-068-3_17. 21468954

[4] Y. N. L. H. Ching , K. M. Meyers , J. A. Brassard , and K. J. Wardrop , “Effect of Cryoprecipitate and Plasma on Plasma von Willebrand Factor Multimeters and Bleeding Time in Doberman Pinschers With Type‐I von Willebrand's Disease,” American Journal of Veterinary Research 55, no. 1 (1994): 102–110. 8141483

[5] R. L. Sparrow , R. J. Simpson , and D. W. Greening , “Preparation of Cryoprecipitate and Cryo‐Depleted Plasma for Proteomic Research Analysis,” Methods in Molecular Biology 2628 (2023): 41–49, 10.1007/978-1-0716-2978-9_4. 36781778

[6] S. F. Idris , A. V. Hadjinicolaou , M. Sweeney , C. Winthrop , G. Balendran , and M. Besser , “The Efficacy and Safety of Cryoprecipitate in the Treatment of Acquired Hypofibrinogenaemia,” British Journal of Haematology 166, no. 3 (2014): 458–461, 10.1111/BJH.12864. 24725203

[7] A. Endo , A. Senda , Y. Otomo , M. Firek , M. Kojima , and R. Coimbra , “Clinical Benefits of Early Concurrent Use of Cryoprecipitate and Plasma Compared With Plasma Only in Bleeding Trauma Patients,” Critical Care Medicine 50, no. 10 (2022): 1477–1485, 10.1097/CCM.0000000000005596. 35759689

[8] T. Stokol and B. W. Parry , “Efficacy of Fresh‐Frozen Plasma and Cryoprecipitate in Dogs With von Willebrand's Disease or Hemophilia A,” Journal of Veterinary Internal Medicine 12, no. 2 (1998): 84–92, 10.1111/j.1939-1676.1998.tb02100.x. 9560764

[9] L. Green , P. Bolton‐Maggs , C. Beattie , et al., “British Society of Haematology Guidelines on the Spectrum of Fresh Frozen Plasma and Cryoprecipitate Products: Their Handling and Use in Various Patient Groups in the Absence of Major Bleeding,” British Journal of Haematology 181, no. 1 (2018): 54–67, 10.1111/BJH.15167. 29527654

[10] P. M. Nair , M. J. Rendo , K. M. Reddoch‐Cardenas , J. K. Burris , M. A. Meledeo , and A. P. Cap , “Recent Advances in Use of Fresh Frozen Plasma, Cryoprecipitate, Immunoglobulins, and Clotting Factors for Transfusion Support in Patients With Hematologic Disease,” Seminars in Hematology 57, no. 2 (2020): 73–82, 10.1053/J.SEMINHEMATOL.2020.07.006. 32892846 PMC7384412

[11] B. Nascimento , L. T. Goodnough , and J. H. Levy , “Cryoprecipitate Therapy,” British Journal of Anaesthesia 113, no. 6 (2014): 922–934, 10.1093/bja/aeu158. 24972790 PMC4627369

[12] J. L. Callum , K. Karkouti , and Y. Lin , “Cryoprecipitate: The Current State of Knowledge,” Transfusion Medicine Reviews 23, no. 3 (2009): 177–188, 10.1016/J.TMRV.2009.03.001. 19539873

[13] H. Wong and N. Curry , “Cryoprecipitate Transfusion: Current Perspectives,” International Journal of Clinical Transfusion Medicine 4 (2016): 89–97, 10.2147/IJCTM.S99042.

[14] C. A. Culler , C. Iazbik , and J. Guillaumin , “Comparison of Albumin, Colloid Osmotic Pressure, von Willebrand Factor, and Coagulation Factors in Canine Cryopoor Plasma, Cryoprecipitate, and Fresh Frozen Plasma,” Journal of Veterinary Emergency and Critical Care 27, no. 6 (2017): 638–644, 10.1111/vec.12671. 29064153

[15] T. Stokol and B. W. Parry , “Stability of von Willebrand Factor and Factor VIII in Canine Cryoprecipitate Under Various Conditions of Storage,” Research in Veterinary Science 59, no. 2 (1995): 152–155, 10.1016/0034-5288(95)90050-0. 8525105

[16] H. Cheung , K. E. Jandrey , J. Burges , M. Brooks , and M. S. Kent , “An In Vitro Study of Canine Cryopoor Plasma to Correct Vitamin K‐Dependent Coagulopathy in Dogs,” Journal of Veterinary Emergency and Critical Care 31, no. 2 (2021): 231–238, 10.1111/vec.13049. 33749109

[17] C. Graf , J. Raila , F. J. Schweigert , and B. Kohn , “Effect of Leukoreduction Treatment on Vascular Endothelial Growth Factor Concentration in Stored Canine Blood Transfusion Products,” American Journal of Veterinary Research 73, no. 12 (2012): 2001–2006, 10.2460/AJVR.73.12.2001. 23176431

[18] R. Locke , D. Paul , S. Touch , A. Mackley , V. Maduskuie , and P. Fawcett , “Cytokine Load in Prestorage Leukoreduced PRBC Transfusions in Premature Infants,” Journal of Perinatology 25, no. 8 (2005): 526–530, 10.1038/sj.jp.7211340. 15908985

[19] J. D. Roback , S. Caldwell , J. Carson , et al., “Evidence‐Based Practice Guidelines for Plasma Transfusion,” Transfusion 50 (2010): 1227–1239, 10.1111/j.1537-2995.2010.02632.x. 20345562

[20] R. Cardigan , J. Sutherland , M. Garwood , et al., “The Effect of Leucocyte Depletion on the Quality of Fresh‐Frozen Plasma,” British Journal of Haematology 114, no. 1 (2001): 233–240, 10.1046/J.1365-2141.2001.02907.X. 11472374

[21] H. S. Alhumaidan , T. A. Cheves Mt , S. Holme , and J. D. Sweeney , “The Effect of Filtration on Residual Levels of Coagulation Factors in Plasma,” American Journal of Clinical Pathology 139, no. 1 (2013): 110–116, 10.1309/AJCPRRESG7PGIAH5. 23270906

[22] O. A. H. Afifi , E. M. N. Abdelsalam , A. A. E. A. M. Makhlouf , and M. A. M. Ibrahim , “Evaluation of Coagulation Factors Activity in Different Types of Plasma Preparations,” Indian Journal of Hematology and Blood Transfusion 35, no. 3 (2019): 551, 10.1007/S12288-018-1043-9. 31388272 PMC6646486

[23] S. Runkel , J. Bach , H. Haubelt , C. Anders , W. Hitzler , and P. Hellstern , “The Impact of Two Whole Blood Inline Filters on Markers of Coagulation, Complement and Cell Activation,” Vox Sanguinis 88, no. 1 (2005): 17–21, 10.1111/J.1423-0410.2005.00591.X. 15663718

[24] M. Heiden , U. Salge , R. Henschler , et al., “Plasma Quality After Whole‐Blood Filtration Depends on Storage Temperature and Filter Type,” Transfusion Medicine 14, no. 4 (2004): 297–304, 10.1111/J.0958-7578.2004.00517.X. 15285726

[25] A. A. A. Enein , H. A. A. Rahman , M. M. M. A. Maged , and M. H. El Sissy , “The Effect of Different Methods of Leucoreduction on Plasma Coagulation Factors,” Blood Coagulation & Fibrinolysis 28, no. 2 (2017): 117–120, 10.1097/MBC.0000000000000548. 28182588

[26] L. Brownlee , K. J. Wardrop , R. K. Sellon , and K. M. Meyers , “Use of a Prestorage Leukoreduction Filter Effectively Removes Leukocytes From Canine Whole Blood While Preserving Red Blood Cell Viability,” Journal of Veterinary Internal Medicine 14, no. 4 (2000): 412–417, 10.1111/J.1939-1676.2000.TB02249.X. 10935891

[27] R. D. Trinder , E. Lo , and K. R. Humm , “The Effects of Leukoreduction on Canine Blood Unit Weight and Processing Time,” Journal of Veterinary Emergency and Critical Care 32, no. 6 (2022): 836–839, 10.1111/VEC.13225. 35712893 PMC9796084

[28] M. T. Antognoni , M. L. Marenzoni , A. L. Misia , et al., “Effect of Leukoreduction on Hematobiochemical Parameters and Storage Hemolysis in Canine Whole Blood Units,” Animals 11, no. 4 (2021): 925, 10.3390/ANI11040925. 33805143 PMC8064101

[29] A. Stefani , K. Capello , A. Carminato , et al., “Effects of Leukoreduction on Storage Lesions in Whole Blood and Blood Components of Dogs,” Journal of Veterinary Internal Medicine 35, no. 2 (2021): 936–945, 10.1111/JVIM.16039. 33591603 PMC7995433

[30] E. Spada , R. Perego , L. Baggiani , and D. Proverbio , “Effect of Leukoreduction by Pre‐Storage Filtration on Coagulation Activity of Canine Plasma Collected for Transfusion,” Veterinary Sciences 8, no. 8 (2021): 157, 10.3390/VETSCI8080157. 34437479 PMC8402778

[31] S. L. Purcell , M. Claus , G. Hosgood , and L. Smart , “Effect of Leukoreduction on Concentrations of Interleukin‐8, Interleukin‐1β, and Tumor Necrosis Factor‐α in Canine Packed Red Blood Cells During Storage,” American Journal of Veterinary Research 76, no. 11 (2015): 969–974, 10.2460/AJVR.76.11.969. 26512542

[32] E. Ergül Ekİz , M. Arslan , I. Akyazi , E. Eraslan Uygur , G. İnal Gültekİn , and M. Özcan , “The Effects of Prestorage Leukoreduction and Storage Duration on the In Vitro Quality of Canine Packed Red Blood Cells,” Turkish Journal of Veterinary and Animal Sciences 36, no. 6 (2012): 711–717, 10.3906/vet-1110-25.

[33] L. A. Lacerda , N. R. C. Hlavac , S. R. Terra , F. P. Back , K. Jane Wardrop , and F. H. D. González , “Effects of Four Additive Solutions on Canine Leukoreduced Red Cell Concentrate Quality During Storage,” Veterinary Clinical Pathology 43, no. 3 (2014): 362–370, 10.1111/VCP.12163. 25135622

[34] M. K. Notomi , R. R. de Gopegui , and P. B. Escodro , “Haematologic Effects of Leukoreduction on Canine Whole Blood Post‐Filtration and Post‐Storage,” Comparative Clinical Pathology 25, no. 1 (2016): 145–149, 10.1007/S00580-015-2155-3 /METRICS.

[35] S. M. Muro , J. H. Lee , J. V. Stokes , et al., “Effects of Leukoreduction and Storage on Erythrocyte Phosphatidylserine Expression and Eicosanoid Concentrations in Units of Canine Packed Red Blood Cells,” Journal of Veterinary Internal Medicine 31, no. 2 (2017): 410–418, 10.1111/JVIM.14664. 28140476 PMC5354049

[36] H. Yang , W. Kim , J. Bae , et al., “Effects of Irradiation and Leukoreduction on Down‐Regulation of CXCL‐8 and Storage Lesion in Stored Canine Whole Blood,” Journal of Veterinary Science 20, no. 1 (2019): 72–78, 10.4142/JVS.2019.20.1.72. 30541183 PMC6351766

[37] M. J. Frank , M. R. Khattab , R. W. Wills , et al., “Effects of Leukoreduction on N‐Methylhistamine Concentration in Stored Units of Canine Whole Blood,” American Journal of Veterinary Research 82, no. 11 (2021): 890–896, 10.2460/AJVR.82.11.890. 34669495

[38] M. L. Foote , M. B. Brooks , T. M. Archer , R. W. Wills , A. J. Mackin , and J. M. Thomason , “Coagulation Factor Activity in Units of Leukoreduced and Nonleukoreduced Canine Fresh‐Frozen Plasma,” American Journal of Veterinary Research 80, no. 9 (2019): 846–851, 10.2460/AJVR.80.9.846. 31449444

[39] M. A. McMichael , S. A. Smith , A. Galligan , K. S. Swanson , and T. M. Fan , “Effect of Leukoreduction on Transfusion‐Induced Inflammation in Dogs,” Journal of Veterinary Internal Medicine 24, no. 5 (2010): 1131–1137, 10.1111/J.1939-1676.2010.0561.X. 20666981

[40] C. A. Culler , A. Balakrishnan , P. E. Yaxley , and J. Guillaumin , “Clinical Use of Cryopoor Plasma Continuous Rate Infusion in Critically Ill, Hypoalbuminemic Dogs,” Journal of Veterinary Emergency and Critical Care 29, no. 3 (2019): 314–320, 10.1111/vec.12834. 31012237

[41] L. Bosch Lozano , S. L. Blois , R. D. Wood , et al., “A Pilot Study Evaluating the Effects of Prestorage Leukoreduction on Markers of Inflammation in Critically Ill Dogs Receiving a Blood Transfusion,” Journal of Veterinary Emergency and Critical Care 29, no. 4 (2019): 385–390, 10.1111/VEC.12857. 31218809

[42] E. B. Davidow , H. Montgomery , and M. Mensing , “The Influence of Leukoreduction on the Acute Transfusion‐Related Complication Rate in 455 Dogs Receiving 730 Packed RBCs: 2014–2017,” Journal of Veterinary Emergency and Critical Care 32, no. 4 (2022): 479–490, 10.1111/VEC.13175. 35043550

[43] S. M. Radulescu , R. Skulberg , C. Mcdonald , D. L. Chan , and K. Humm , “Randomized Double‐Blinded Clinical Trial on Acute Transfusion Reactions in Dogs Receiving Leukoreduced Versus Nonleukoreduced Packed Red Blood Cells,” Journal of Veterinary Internal Medicine 35, no. 3 (2021): 1325–1332, 10.1111/jvim.16138. 33960540 PMC8162603

[44] M. A. Claus , D. Poh , L. Smart , S. L. Purcell , C. J. Boyd , and C. R. Sharp , “Effect of Leukoreduction on Inflammation in Critically Ill Dogs Receiving Red Blood Cell Transfusions: A Randomized Blinded Controlled Clinical Trial,” Journal of Veterinary Internal Medicine 36, no. 4 (2022): 1248–1257, 10.1111/JVIM.16487. 35792764 PMC9308429

[45] Ministero Della Salute Dipartimento Della Sanità Pubblica e Dell'Innovazione , “Linea Guida Relativa All'Esercizio delle Attività Riguardanti la Medicina Trasfusionale in Campo Veterinario,” 2016, 25: 5–18, https://www.trovanorme.salute.gov.it/norme/dettaglioAtto?id=54057.

[46] J. Riggert , D. W. M. Schwartz , J. U. Wieding , W. R. Mayr , and M. Köhler , “Prestorage Inline Filtration of Whole Blood for Obtaining White Cell‐Reduced Blood Components,” Transfusion 37, no. 10 (1997): 1039–1044, 10.1046/J.1537-2995.1997.371098016442.X. 9354822

[47] J. Riggert , G. Simson , J. Dittmann , and M. Köhler , “Prestorage Leukocyte Depletion With In‐Line Filtration of Whole Blood in Comparison With Blood Component Leukocyte Depletion,” Vox Sanguinis 69, no. 3 (1995): 201–205, 10.1111/J.1423-0410.1995.TB02594.X. 8578731

[48] A. Rapaille , G. Moore , J. Siquet , J. Flament , and D. Sondag‐Thull , “Prestorage Leukocyte Reduction With In‐Line Filtration of Whole Blood: Evaluation of Red Cells and Plasma Storage,” Vox Sanguinis 73, no. 1 (1997): 28–35, 10.1046/J.1423-0410.1997.7310028.X. 9269067

[49] L. M. Williamson , J. R. Rider , I. D. Swann , M. A. Winter , F. Ali , and D. H. Pamphilon , “Evaluation of Plasma and Red Cells Obtained After Leucocyte Depletion of Whole Blood,” Transfusion Medicine 9, no. 1 (1999): 51–61, 10.1046/J.1365-3148.1999.009001051.X. 10216905

[50] B. G. Solheim , O. Flesland , F. Brosstad , T. E. Mollnes , and J. Seghatchian , “Improved Preservation of Coagulation Factors After Pre‐Storage Leukocyte Depletion of Whole Blood,” Transfusion and Apheresis Science 29, no. 2 (2003): 133–139, 10.1016/S1473-0502(03)00117-4. 12941351

[51] K. S. K. Chan and R. L. Sparrow , “Microparticle Profile and Procoagulant Activity of Fresh Frozen Plasma Is Affected by Whole Blood‐Leukocyte Depletion Rather Than 24‐Hour Room Temperature‐Hold,” Transfusion 54, no. 8 (2014): 1935, 10.1111/TRF.12602. 24635475 PMC4164532

[52] D. Avenick , L. Kidd , S. Istvan , et al., “Effects of Storage and Leukocyte Reduction on the Concentration and Procoagulant Activity of Extracellular Vesicles in Canine Packed Red Cells,” Journal of Veterinary Emergency and Critical Care 31, no. 2 (2021): 221–230, 10.1111/VEC.13050. 33751799

[53] E. Kretzschmar , F. Kruse , O. Greiss , D. Paunovic , T. Kallweit , and H. Trobisch , “Effects of Extended Storage of Whole Blood Before Leucocyte Depletion on Coagulation Factors in Plasma,” Vox Sanguinis 87, no. 3 (2004): 156–164, 10.1111/J.1423-0410.2004.00563.X. 15569067

[54] S. V. Rudmann , Textbook of Blood Banking and Transfusion Medicine, 2nd ed., ed. S. V. Rudman (Elsevier Saunders, 2005).

[55] E. R. Burka , T. Puffer , and J. Martinez , “The Influence of Donor Characteristics and Preparation Methods on the Potency of Human Cryoprecipitate,” Transfusion 15, no. 4 (1975): 323–328, 10.1046/j.1537-2995.1975.15476034551.x. 1166505

[56] C. K. Kasper , B. A. Myhre , J. D. McDonald , Y. Nakasako , and D. I. Feinstein , “Determinants of Factor VIII Recovery in Cryoprecipitate,” Transfusion 15, no. 4 (1975): 312–322, 10.1046/j.1537-2995.1975.15476034550.x. 1166504

[57] M. Freedman and G. Rock , “Analysis of the Products of Cryoprecipitation: RiCoF Is Deficient in Cryosupernatant Plasma,” Transfusion and Apheresis Science 43, no. 2 (2010): 179–182, 10.1016/J.TRANSCI.2010.07.004. 20719565

[58] J. Duguid , D. F. O'Shaughnessy , C. Atterbury , et al., “Guidelines for the Use of Fresh‐Frozen Plasma, Cryoprecipitate and Cryosupernatant,” British Journal of Haematology 126, no. 1 (2004): 11–28, 10.1111/J.1365-2141.2004.04972.X. 15198728

[59] Y. Hori , M. Hayakawa , A. Isonishi , K. Soejima , M. Matsumoto , and Y. Fujimura , “ADAMTS13 Unbound to Larger von Willebrand Factor Multimers in Cryosupernatant: Implications for Selection of Plasma Preparations for Thrombotic Thrombocytopenic Purpura Treatment,” Transfusion 53, no. 12 (2013): 3192–3202, 10.1111/TRF.12182. 23560518

[60] E. A. Tseǐmakh , V. A. Bombizo , P. N. Buldakov , et al., “The Use of Cryosupernatant Plasma in Complex Treatment of Pancreonecrosis,” Khirurgiia (Sofiia), no. 8 (2008): 32–37. 18833146

[61] E. A. Tseimakh , S. A. Kundius , V. A. Bombizo , et al., “Comparative Data About Cryosupernatant and Fresh Frozen Plasma Use in Treatment of Disseminated Intravascular Coagulation in Patients With Generalized Peritonitis,” Anesteziologiia i Reanimatologiia, no. 2 (2014): 52–56. 25055495

[62] A. M. Bentley , C. M. Otto , and F. S. Shofer , “Comparison of Dogs With Septic Peritonitis: 1988–1993 Versus 1999–2003: Retrospective Study,” Journal of Veterinary Emergency and Critical Care 17, no. 4 (2007): 391–398, 10.1111/J.1476-4431.2007.00251.X.

[63] E. M. Mazzaferro , E. Rudloff , and R. Kirby , “The Role of Albumin Replacement in the Critically Ill Veterinary Patient,” Journal of Veterinary Emergency and Critical Care 12, no. 2 (2002): 113–124, 10.1046/J.1435-6935.2002.00025.X.

[64] E. M. Craft and L. L. Powell , “The Use of Canine‐Specific Albumin in Dogs With Septic Peritonitis,” Journal of Veterinary Emergency and Critical Care (San Antonio, Tex.) 22, no. 6 (2012): 631–639, 10.1111/J.1476-4431.2012.00819.X. 23216837

[65] S. Adamantos , D. L. Chan , R. Goggs , and K. Humm , “Risk of Immunologic Reactions to Human Serum Albumin Solutions,” Journal of Small Animal Practice 50, no. 4 (2009): 206, 10.1111/J.1748-5827.2009.00752.X. 19320816

[66] E. Mazzaferro and L. L. Powell , “Fluid Therapy for the Emergent Small Animal Patient: Crystalloids, Colloids, and Albumin Products,” Veterinary Clinics of North America. Small Animal Practice 43, no. 4 (2013): 721–734, 10.1016/J.CVSM.2013.03.003. 23747257

[67] D. K. McRoyan , C. J. McRoyan , K. L. Sauter , P. I. Liu , and S. J. Daniel , “Antithrombin III, Plasminogen, Plasmin, and Alpha‐2‐Antiplasmin in Donor Blood and Plasma Components,” Annals of Clinical and Laboratory Science 15, no. 2 (1985): 165–170. 3158265

[68] J. Ichikawa , T. Iba , R. Okazaki , et al., “Hemostatic Capability of Ultrafiltrated Fresh Frozen Plasma Compared to Cryoprecipitate,” Scientific Reports 13, no. 1 (2023): 21579, 10.1038/S41598-023-48759-1. 38062086 PMC10703847

[69] P. D. Mintz , P. M. Blatt , W. J. Kuhns , and H. R. Roberts , “Antithrombin III in Fresh Frozen Plasma, Cryoprecipitate, and Cryoprecipitate‐Depleted Plasma,” Transfusion 19, no. 5 (1979): 597–598, 10.1046/J.1537-2995.1979.19580059818.X. 505534

[70] T. Chatterjee , J. Philip , V. Nair , et al., “Inherited Factor X (Stuart–Prower Factor) Deficiency and Its Management,” Medical Journal, Armed Forces India 71 (2015): S184–S186, 10.1016/J.MJAFI.2014.01.007. 26265825 PMC4529547

[71] E. P. Soundar , M. Reyes , L. Korte , and A. Bracey , “Characteristics of Thawed Pooled Cryoprecipitate Stored at Refrigerated Temperature for 24 Hours,” Blood Transfusion 16, no. 5 (2018): 443–446, 10.2450/2017.0133-17. 29106354 PMC6125233

[72] C. Thomson , M. Sobieraj‐Teague , D. Scott , E. Duncan , S. Abraham , and D. Roxby , “Extending the Post‐Thaw Viability of Cryoprecipitate,” Transfusion 61, no. 5 (2021): 1578–1585, 10.1111/TRF.16366. 33728705

[73] J. L. Fenderson , M. A. Meledeo , M. J. Rendo , et al., “Hemostatic Characteristics of Thawed, Pooled Cryoprecipitate Stored for 35 Days at Refrigerated and Room Temperatures,” Transfusion 59, no. S2 (2019): 1560–1567, 10.1111/TRF.15180. 30980741

[74] P. E. Yaxley , M. W. Beal , L. A. Jutkowitz , et al., “Comparative Stability of Canine and Feline Hemostatic Proteins in Freeze‐Thaw‐Cycled Fresh Frozen Plasma,” Journal of Veterinary Emergency and Critical Care 20, no. 5 (2010): 472–478, 10.1111/j.1476-4431.2010.00563.x. 20955297

[75] C. L. Mansell and M. Boller , “Blood Component Processing and Storage,” in Manual of Veterinary Transfusion Medicine and Blood Banking, 1st ed., ed. K. Yagi and M. K. Holowaychuk (John Wiley & Sons, Inc, 2022), 237–255.

[76] A. C. G. Abrams Ogg and S. L. Blois , “Principles of Canine and Feline Blood Collection, Processing, and Storage,” in Schalm's Veterinary Hematology, 7th ed., ed. M. B. Brooks , K. E. Harr , D. M. Seelig , K. J. Wardrop , and D. J. Weiss (John Wiley & Sons, Inc, 2022), 898–907.

